# Generational Effects of Opioid Exposure

**DOI:** 10.3390/encyclopedia1010012

**Published:** 2021-01-18

**Authors:** Katherine E. Odegaard, Gurudutt Pendyala, Sowmya V. Yelamanchili

**Affiliations:** Department of Anesthesiology, University of Nebraska Medical Center, Omaha, NE 68198, USA

**Keywords:** opioids, morphine, heroin, oxycodone, multigenerational, intergenerational, transgenerational, humans, animals

## Abstract

The inheritance of substance abuse, including opioid abuse, may be influenced by genetic and non-genetic factors related to the environment, such as stress and socioeconomic status. These non-genetic influences on the heritability of a trait can be attributed to epigenetics. Epigenetic inheritance can result from modifications passed down from the mother, father, or both, resulting in either maternal, paternal, or parental epigenetic inheritance, respectively. These epigenetic modifications can be passed to the offspring to result in multigenerational, intergenerational, or transgenerational inheritance. Human and animal models of opioid exposure have shown generational effects that result in molecular, developmental, and behavioral alterations in future generations.

## 1. Introduction

The ongoing opioid epidemic in the United States and worldwide is characterized by an increase in use of licit and illicit opioid substances, such as prescription opioids, illicit heroin, and illicitly-produced fentanyl. While steps have been taken by the FDA to limit opioid abuse and misuse, the potential for addiction remains an issue, especially when individuals turn to cheaper, illicit opiates as an alternative to prescription opioids [1]. Further, opioids are commonly prescribed to postpartum women, often in abundance [2,3], making pregnant women a particularly vulnerable group in the opioid epidemic. Additionally, mounting evidence suggests that substance use disorders, such as opioid abuse, run in families [4,5]; twin, adoption, and sibling studies implicate genetic factors are involved in the heritability of substance abuse [6]. Several human genome-wide association studies have been done to identify loci and genes associated with addiction and substance use disorders [5,7,8], revealing heritability estimates of substance use between 0.39 and 0.72 [7]. However, the inheritance of substance abuse cannot be explained entirely through genetic mechanisms alone. Environmental factors, such as stress and socioeconomic status, also shape an individual’s susceptibility to addiction [9].

### 1.1. Generational Inheritance

These non-genetic influences on the heritability of a trait can be attributed to epigenetics. Epigenetics is the alteration of gene expression without changes to DNA sequence [10,11], which can be accomplished through acetylation or methylation of the histone complexes [12]. Epigenetic inheritance can result from modifications passed down from the mother, father, or both, resulting in either maternal, paternal, or parental epigenetic inheritance, respectively. Because these epigenetic modifications can be passed to the offspring, it is important to define the type of generational inheritance: multigenerational, intergenerational, or transgenerational [13].

Skinner has defined transgenerational inheritance as “germ-line-mediated inheritance of epigenetic information between generations in the absence of direct environmental influences that lead to phenotypic variation,” meaning a true transgenerational study must include at least one generation that receives no direct exposure to the stimulus [14]. While the definition of transgenerational inheritance is fairly straightforward, defining inter-and multi-generational inheritance is slightly more difficult. Vassoler et al. provide the simplest definitions for inter- and multi-generational inheritance [13]. If the F0 drug use occurs in males or in females prior to pregnancy, the germ cells, which will become the F1 generation, are exposed to the drug. Because both the F0 and the F1 generations are directly exposed to the drug, their inheritance pattern is considered intergenerational. In this scenario, the F2 generation would be the first generation not exposed to the drug, resulting in transgenerational inheritance. Alternatively, if the F0 female is exposed to the drug during pregnancy or postpartum, the somatic and germ cells of the F1 offspring receive direct exposure to the drug in utero or postpartum via the breastmilk [12]. Since the F1 germ cells are exposed to the drug, and the F2 offspring originate from those germ cells, the first generation without direct exposure to the drug is F3. In these cases, a study spanning from F0 to F3 would be considered transgenerational, as F3 is the first generation without direct exposure; a study spanning from F0 to F2 would be considered multigenerational.

**Figure 1.**
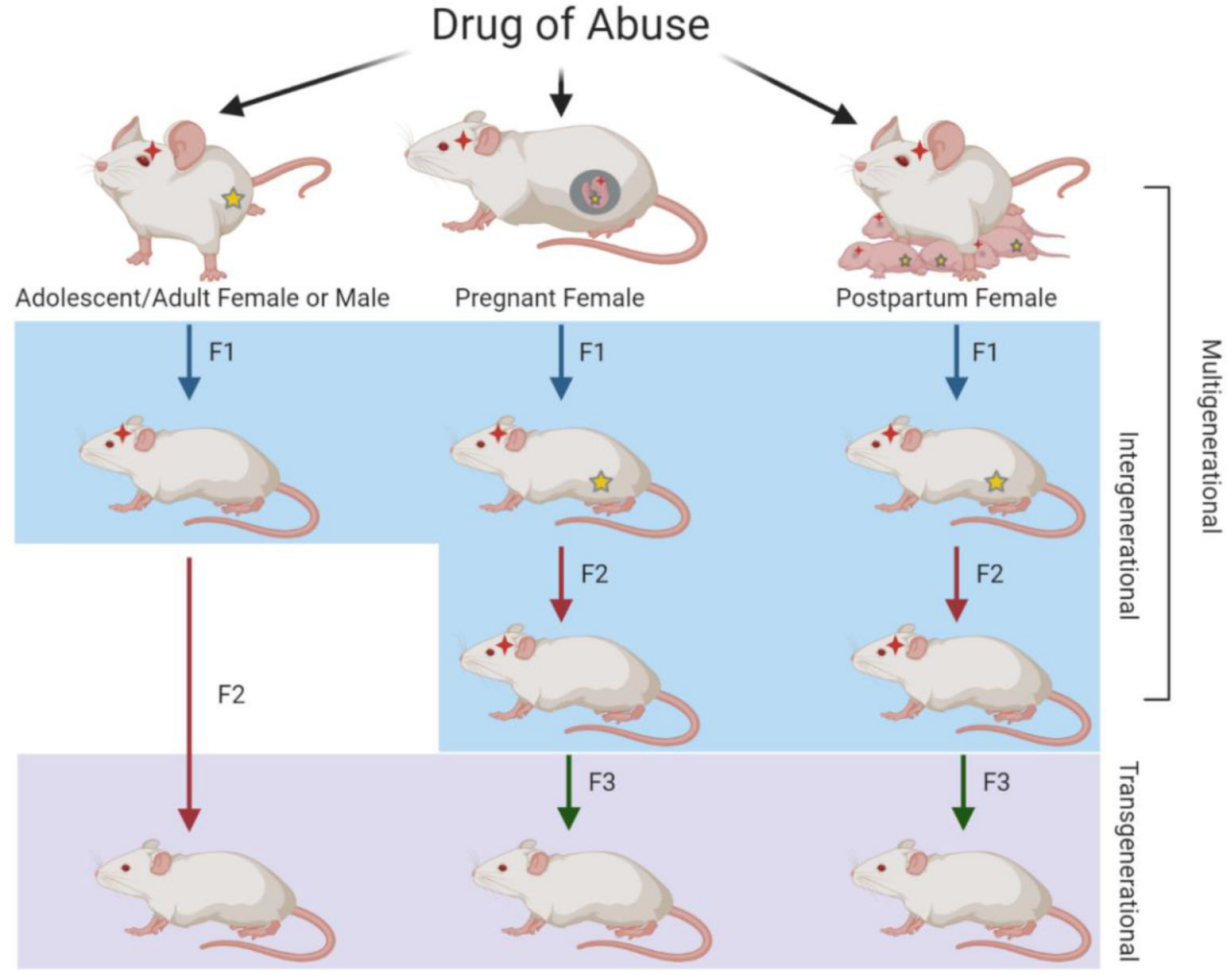
Illustration of multi-, inter-, and trans-generational relationships resulting from parental drug exposure. When F0 males or females are exposed to drugs of abuse in adolescence or adulthood, the germ cells that give rise to the F1 generation are directly exposed, resulting in intergenerational transmission. Because the germ cells of the F1 generation were not directly exposed, the F2 generation represents transgenerational transmission. When F0 pregnant or postpartum breastfeeding females are exposed, the F1 generation and their germ cells that will give rise to the F2 generation are also directly exposed, resulting in intergenerational transmission. For both of these scenarios, the F3 generation is the first without direct drug exposure and therefore represents transgenerational transmission. As F0–F2 generations have direct drug exposure in the case of pregnant or postpartum females, the relationship among the three generations represents multigenerational transmission. Red stars represent direct exposure to that particular generation while yellow stars represent direct exposure to the germ cells.

Hanson et al. have outlined the definition of multigenerational inheritance as “co-incident direct exposure of multiple generations to an environmental factor promoting alterations in the multiple generations exposed” [15]. Therefore, in the scenario of drug exposure during F0 pregnancy, the relationship among F0, F1, and F2 would be multigenerational, as the effects of direct exposure to the drug span more than two generations. The paired relationships between F0–F1 and F1–F2, however, would be intergenerational, as these pairs span only two generations post-direct drug exposure. A depiction of multi-, inter-, and trans-generational types of inheritance are shown in Figure 1.

With regard to generational studies and opioid exposure, studies involving in utero exposure to opioids are particularly critical, as these may result in neonatal opioid withdrawal syndrome (NOWS) or neonatal abstinence syndrome (NAS). Neonates with NOWS/NAS exhibit high-pitched cries, tremors, difficulty feeding, hypertonia, and breathing issues [16]. In the context of in utero opioid exposure and NOWS/NAS, the fetal origins of adult disease hypothesis, first posited by Dr. David Barker in 1995 [17], is particularly interesting. This hypothesis states that risk factors from intrauterine environmental exposures affect fetal development during sensitive periods, and increase the risk of specific diseases in adult life [18]. Indeed, prenatal exposure to opioids has been associated with smaller head size, lighter birthweights, and shorter body lengths in neonates [19–24]. Moreover, not only do young adults exposed to heroin prenatally exhibit cognitive and motor function deficits [25,26], but they may also have an 8-fold increased risk of depression, a 3-fold increased risk of attention disorders, and a 16-fold increased risk of substance use disorders [27]. As substance use disorder is considered a disease, it is critical to understand how the generational effects of opioid exposure manifest in not only newborns, but adults as well.

This review aims to discuss the generational effects of opioid exposure in animal and human studies. The following sections will provide a brief overview of opioids and discuss the molecular and behavioral changes reported in exposed generations and, where applicable, whether these changes are present long-term. As the opioid epidemic continues, understanding the generational impacts and long-term effects of opioid exposure is paramount.

### 1.2. Opioids

Opiate analgesics have a long history of clinical use in the treatment of chronic pain. First derived from opium in 1803 [28], morphine became widely used with the technological innovation of the perfected hypodermic syringe in 1853, which allowed for faster delivery of morphine into the blood or tissue. The American Civil War (1861–1865), the Prussian–Austrian War (1866), and the Franco–Prussian War (1870) also rapidly increased the use of morphine for the reduction of pain and relief from dysentery, often leading to a dependence later deemed “soldier’s disease” or the “army disease” [29]. In England, morphine was first recommended for treatment of cancer pain in the 1950s [30]. Taken orally or by injection, morphine is still widely used today for acute and chronic pain management. Unfortunately, opioids are also used illicitly and often taken in excess, affecting the behavior of the user[31].

Whether used licitly or illicitly, opioids activate three types of receptors (mu, MOR; delta, DOR; kappa, KOR) in the dopaminergic system. The nucleus accumbens (NAc) is largely affected and undergoes changes in density of the dendritic spines [31], effectively altering the plasticity of the dendritic spines during nervous system development. This neural plasticity is critical in the development of addiction and other ingrained behaviors. The increased abuse of prescription and non-prescription opioids has resulted in a severe public health crisis throughout large swaths of America [32–34]. In 2017, over two-thirds of drug-overdose deaths resulted from opioid abuse [35], and opioid overdose-attributed deaths have tripled since the turn of the new millennium [36]. The main opioids discussed in this review are morphine, heroin, and oxycodone.

#### 1.2.1. Morphine

Morphine is a potent analgesic primarily used in hospitals to combat severe pain. While morphine may be effective for pain relief, there are potential adverse side effects such as tolerance and addiction as well as molecular alterations [37]. Morphine’s powerful analgesic effects stem from its role as an opioid receptor agonist. By binding to MOR and KOR, morphine blocks the transmission of nociceptive signals, signals pain-modulating neurons in the spinal cord, and inhibits primary afferent nociceptors to the dorsal horn sensory projection cells [38]. Morphine also activates the reward pathway by binding to receptors in the ventral tegmental area (VTA) and NAc, leading to an influx of dopamine in the synapse [39].

#### 1.2.2. Heroin

The result of a chemical modification to morphine, heroin is about three times as potent as its parent drug [29]. Unlike morphine, which can be prescribed, heroin is an illicit substance. Like other opioids, heroin acts agonistically to the three opioid receptors. A particular danger in heroin use is that heroin often contains additives that may clog blood vessels leading to the lungs, liver, kidneys, or brain, causing permanent damage. Needle sharing and impaired judgment resulting from heroin use can also increase the risk of contracting infectious diseases such as HIV and hepatitis [40].

#### 1.2.3. Oxycodone

Structurally similar to morphine, oxycodone has been established as a potent and widely abused prescription opioid. Designed for pain management, oxycodone operates primarily as a full agonist to MOR [41]. The binding of oxycodone to MOR subsequently inhibits neurotransmitter release by decreasing cAMP production [41]. Importantly, oral doses of extended release oxycodone are thought to be twice as potent as a similar dose of morphine [42], contributing to its popularity and abuse.

## 2. Animal Studies

Several animal studies using rats and mice have been conducted to determine multi-, inter-, and transgenerational effects of opioid exposure, with most studies being conducted using morphine, heroin, and oxycodone. The studies discussed in this section involve maternal, paternal, parental, and perinatal exposure paradigms. Maternal and paternal studies were conducted in adolescent and adult models. Maternal models are also part of the perinatal paradigms as the opioids were administered to the mother and passed to the offspring through the placenta (in utero exposure) or via the breastmilk (postnatal exposure). In parental paradigms, both parents were exposed to opioids.

### 2.1. Morphine

A number of studies using rats have been conducted to determine the generational effects of parental morphine use on offspring. Chronic morphine exposure during adolescence has been shown to significantly impact male reproductive parameters, as evidenced by reduced seminal vesicle and testes weights [43], and delay sexual maturation [44]. Additionally, offspring born to males with adolescent morphine exposure had several endocrine deficits in adulthood. Specifically, adult male offspring born to morphine-treated males had lower serum testosterone and luteinizing hormone levels and higher adrenal weights [43]. In female offspring, serum corticosterone and β-endorphin levels were significantly higher, but no differences in reproductive endocrine status were observed. Interestingly, F1 and F2 males descended from adolescent morphine-exposed dams had blunted corticosterone secretion, an effect that was specific to offspring from females exposed to morphine during adolescence as opposed to those exposed during adulthood [45]. These males also had significantly lower levels of paraventricular nucleus (PVN) corticotropin releasing hormone (CRH). F2 males also exhibited dysregulated MOR expression in the PVN [45]. Interestingly, on a fat–sugar diet (FSD), F1 males born to adolescent morphine-exposed females had increased weight gain, fasting glucose, insulin, and corticosterone levels, as well as increased neuropeptide Y (NPY), a potent orexigen [44]. Maternal opioid exposure in adolescence appears to interact with F1 offspring diet to further disrupt hypothalamic and neuroendocrine systems that influence sexual maturation, energy metabolism, synaptic plasticity, and possibly immune function [44].

Interestingly, dams with adolescent morphine exposure were shown to have reduced levels of prolactin secretion postpartum, suggesting that morphine use during the pubertal period may affect prolactin secretion into adulthood, potentially affecting the maternal behavior and subsequent behaviors of the offspring [46]. Indeed, Johnson et al. found that adolescent exposure to morphine did affect maternal behavior, with exposed mothers spending less time nursing and grooming pups and more time away from the nest and engaging in self-directed behavior [47]. Perhaps as a result of maternal effects, female offspring of exposed dams exhibited increased anxiety-like behavior [48,49]. Further, male and female offspring appeared to develop a tolerance for the sedative effects of morphine more quickly than offspring born to untreated dams [48,49]. Male offspring also demonstrated decreased rough and tumble play behaviors, while females exhibited a tendency toward increased rough and tumble play, demonstrating sex-specific effects of maternal adolescent morphine exposure on the F1 generation [47]. To determine changes in the reward system, Vassoler et al. used conditioned place preference (CPP) and locomotor sensitization. F1 animals showed increased sensitivity to morphine, and females showed increased locomotor activity when compared to males [50]. Additionally, all F1 animals born to morphine-exposed dams had decreased MOR protein expression in the VTA, while only females had an increased expression of MOR in the NAc. This differential expression of MOR in the NAc and VTA may contribute to the increased sensitivity to morphine seen in these offspring born to morphine-exposed mothers [50].

Transgenerational effects of maternal adolescent morphine exposure have also been reported. Male offspring of morphine-exposed dams had previously shown enhanced locomotor sensitization in response to repeated morphine exposure [48]. As dopamine agonists have been shown to affect locomotor sensitization, Byrnes et al. sought to investigate the effect of quinpirole, a dopamine D2/D3 receptor agonist, on locomotor sensitization in F1 and F2 male progeny of morphine-exposed F0 dams [51]. Quinpirole treatment resulted in an increased locomotor response and also increased plasma corticosterone levels in both generations. Repeated administration of quinpirole resulted in increased expression of KOR and dopamine D2 receptor in the NAc of the males in both generations. Together, these data may suggest the presence of functional and transgenerational neuroadaptations within the NAc that are transmitted across two generations following maternal adolescent exposure to opiates [51].

The effects of adolescent morphine exposure are not limited to females, however. Paternal, maternal, and parental adolescent morphine exposure have been shown to increase anxiety-like behavior in male offspring, depressive-like behavior in female offspring, and enhanced morphine consumption in both sexes during adolescence [52]. Interestingly, parental exposure to an enriched environment reduced anxiety- and depressive-like behaviors and voluntary morphine consumption in the offspring, offering a potential preventative measure in the development of these traits in future offspring [52]. Further, paternal adolescent morphine exposure also altered pain perception [53] and several electrophysiological properties in the locus coeruleus of male progeny [54]. Additionally, F0 male and female parents chronically exposed to morphine in adolescence had increased levels of hippocampal TNF-α, an inflammatory cytokine; F1 male and female offspring of these parents showed the same increase in hippocampal TNF-α [55]. Fathers, but not mothers, also had lower levels of S100B, another key protein in neuroinflammation. This decrease in S100B was also reported in the male and female offspring, suggesting that adult exposure to morphine can induce hippocampal modifications intergenerationally [55]. Additionally, males born to morphine-exposed fathers exhibited morphine tolerance and decreased firing of VTA dopamine neurons [56].

Adolescent morphine exposure in males may also impact future substance abuse of the F1 generation. Male and female F1 progeny have delayed acquisition and decreased intake of cocaine. In addition, they have blunted self-administration levels compared to control rats. Female offspring also had increased levels of morphine intake during acquisition and increased self-administration for oxycodone [57]. Surprisingly, even following acquisition of morphine self-administration, males and females still demonstrated a blunted effort for cocaine. Brain-derived neurotrophic factor (BDNF), an important factor in hippocampal synaptic plasticity, neurogenesis, and modulation of learning and memory, was increased in the prefrontal cortex (PFC) in F1 male and female offspring. Together, these data identify systems that are vulnerable to the adolescent parental exposure and how they may impact drug use of future generations.

Prenatal morphine exposure studies in animals have offered insight into the developmental effects on learning and memory. Prenatal morphine exposure has been shown to affect hippocampal neuron viability and expression of proteins crucial for synaptic plasticity. Increased hippocampal neuron apoptosis, decreased CaMKII (Ca(2+)/calmodulin-dependent kinase II; essential for the induction of synaptic potentiation and memory formation) activity, and decreased BDNF expression have been reported in offspring of prenatally exposed to morphine [58,59]. Prenatal morphine exposure has also been shown to decrease post-synaptic density protein 95 (PSD-95; implicated in the regulation of synaptogenesis, synaptic plasticity, learning and memory) expression in the hippocampus [60,61]. Prenatal exposure also reduced the magnitude of the expression of nitric oxide synthase (*n*NOS), long-term depression (LTD; a mechanism of learning and memory), and phosphorylated CREB^Serine–133^ (an important transcription factor for mammalian learning and memory) in the hippocampal CA1 region of post-natal day 14 (P14) pups. Interestingly, prenatal coadministration of dextromethorphan, an antitussive drug that has been shown to mitigate morphine tolerance and withdrawal [62,63], during pregnancy and throughout lactation significantly attenuated these changes in the hippocampus, presenting a potential therapeutic strategy for combatting these effects of prenatal morphine exposure [60].

Intriguingly, another cellular process underlying learning and memory, long-term potentiation (LTP) has also been reported to be reduced in offspring prenatally exposed to morphine [59,64–66]. Niu et al. suggested that depressed LTP in the morphine group may denote a change in GABAergic inhibition; indeed, a loss of GABA-containing neurons in the dentate gyrus (DG) area of the morphine group was observed [65]. Subsequent behavioral tests to elucidate the impact of these molecular changes have shown altered long- and short-term learning and memory tasks [58,60], impaired working and cued reference memory [61], impaired spatial learning and memory [59,65,67], and enhanced maintenance but impaired acquisition of contextual fear memory [66] in rats prenatally exposed to morphine. Further, prenatally exposed offspring exhibited reduced anxiety-like behavior and no differences in locomotor activity [66], contrary to what was reported in the adolescent morphine studies mentioned previously. Intriguingly, postnatal exercise and enriched environment was shown to improve deficits in spatial learning, hippocampal LTP, and BDNF levels caused by prenatal morphine exposure [59].

Adult parental exposure prior to pregnancy and gestation has also been shown to affect behavior and hippocampal signaling. F0 males and females exposed to morphine as adults yielded F1 offspring that exhibited anxiety-like behavior [68,69] and dendritic retraction in the DG of the hippocampus in adulthood [69]. Further, CRH levels in the plasma and CSF (cerebrospinal fluid), along with CRH receptor 1 mRNA levels, were also increased in F1 progeny while protein kinase C (PKC) levels were decreased [68]. Additionally, insulin-like growth factor (IGF)-2 signaling in the granular zone of the DG was downregulated in these F1 animals. Interestingly, overexpression of IGF-2 prevented anxiety-like behaviors in these offspring. Further, exposure to an enriched environment during adolescence corrected the reduction of hippocampal IGF-2 expression, normalized anxiety-like behavior, and reversed dendritic retraction in the adult offspring [69]. This study suggests that IGF-2 and an enriched environment may be potential forms of intervention to prevent anxiety and brain atrophy in the offspring of parental opioid exposure.

Further, males born to parents with adult morphine exposure exhibited significant memory impairments and tolerance to morphine that were more prominent with maternal morphine exposure than either paternal or parental exposure [70]. Additionally, maternal adult morphine exposure resulted in diminished spatial memory in males in the F1 and F2 generations [71]. Hippocampal genes *Mecp2*, which provides instructions for a transcriptional repressor, and *Hdac2*, which codes for a histone deacetylase important in cognitive function, were also significantly upregulated in the males of both generations [71]. These changes were not seen in the female F1 and F2 progeny, suggesting sex-specific alterations resulting from maternal adult morphine exposure. Ellis et al. have also shown that paternal morphine exposure produces sex-specific impairments in object recognition memory in female offspring [72]. Additionally, offspring born to parents with adult morphine exposure had decreased object recognition, possibly due to decreased histone H3 acetylation and ΔFosB levels in the PFC and hippocampus [73]. Interestingly, nociception was reduced in offspring born to adult morphine-exposed parents, and these offspring also had a low neuronal firing rate and enhanced opioid receptor expression in the NAc [74].

Furthermore, F1 male progeny of morphine-exposed parents had a greater preference for morphine and more anxiety-like behavior; these effects were not seen in the F2 generation [75]. F1 male progeny also exhibited increased expression of D1 and D5 dopamine receptors in the PFC and NAc; D5 and D2 receptors were decreased in the hippocampus. The D4 dopamine receptor was augmented in striatum and hippocampus and decreased in the PFC [75]. Alterations in dopamine receptor expression within the reward system may be one mechanism responsible for these behavioral changes in F1 offspring. Interestingly, injection of SCH23390, a dopamine D1 receptor antagonist, into the PFC and the hippocampus improved spatial memory that was previously reported to be impaired in males born to parents with adult morphine exposure [76].

### 2.2. Heroin

Rat models of parental heroin exposure have provided insight into how heroin exposure in the F0 generation can affect subsequent generations. In one transgenerational study of adolescent paternal exposure, F0 male exposure resulted in smaller F1 litter sizes as well as heightened anxiety and increased aggression in both F1 and F2 offspring; these effects appeared to subside in the F3 generation [77]. While paternal heroin exposure has only recently been explored, several studies of prenatal heroin exposure have been done in mice models. For example, Zhu et al. found that heroin-exposed pups had a marked reduction in birth weight, and the postnatal weight gain in these pups was significantly lower than that of controls, particularly in female pups [78]. These female pups also showed a significant increase in ambulation and rearing, suggesting that prenatal heroin exposure could result in a sex-specific delay of postnatal development and learning [78].

With regard to learning, prenatal heroin exposure has been shown to affect the hippocampus and memory-associated behaviors. Prenatal exposure to heroin in mice resulted in increased expression of hippocampal *caspase-3* and *Bax* and decreased expression of *Bcl*-2, which together may enhance neuronal apoptosis and impair hippocampus-dependent learning and memory [79]. Interestingly, prenatal heroin exposure was also shown to decrease dendrite length and branch number in the somatosensory cortex [80]. Further, mice prenatally exposed to heroin displayed behavioral deficits in eight-arm and Morris mazes [79,81] and exhibited impaired short-term spatial memory as adults [80]. These mice also had increased synaptic activity in cholinergic hippocampal cells [81]. Additional research found that these hippocampus-related behavioral deficits may be linked to the cholinergic receptor-mediated translocation of PKC isozyme PKCγ, which has a role in learning and memory [82–84]. Interestingly, the alterations in cell signaling and subsequent behavior deficits found in prenatal mice models were also reported with pre-hatch heroin exposure in chickens, where heroin exposure affected imprinting behavior and PKCγ [81].

### 2.3. Oxycodone

Several studies of perinatal oxycodone have been conducted in rats to deduce the negative effects of oxycodone exposure in utero or postnatally. One study of prenatal oxycodone exposure found that rat pups exposed in utero had congenital malformations of the face, mouth, and vertebrae as well as intrauterine growth retardation [85]. In adolescence, these animals had lower body and kidney weights. Additionally, endothelin receptor A expression was higher at P1 but appeared to return to baseline by P7, P14, and P28. Conversely, endothelin receptor B expression was lower in the exposed offspring at P1 and P7, returned to baseline at P14, and was increased by P28. As the endothelin receptors are important in normal development of the central nervous system (CNS), the alterations in their expression levels in these offspring may suggest a delay in CNS development [85].

Additionally, prenatal oxycodone exposure rat models have shown alterations in cardiovascular responses as well as behavior. Rats exposed to oxycodone in utero showed subtle alterations in stress cardiovascular response that subsided with age. In adulthood, these rats exhibited impaired memory of stress conditioning learned with adolescent tail shock testing, suggesting the presence of a memory deficit associated with prenatal oxycodone exposure [86]. Indeed, prenatal oxycodone exposure has been shown to impair spatial learning and memory [87]. Prenatal oxycodone exposure rats made a greater number of reference memory errors in the beginning of radial arm maze testing. They also had a deficit in memory retention when assessed by T-maze five days post-acquisition training. In Morris water maze, prenatally exposed rats exhibited poor acquisition during long inter-trial intervals only, with no deficit reported during short inter-trial intervals. Exposed rats also had an increased latency to find and greater distance traveled to the platform in the Morris water maze [87]. Using several spatial memory tasks, this study by Davis et al. indicated that prenatal oxycodone exposure consistently impairs learning and memory [87]. Interestingly, other behavioral paradigms have linked prenatal oxycodone exposure and hyperactivity in adult rats, which is consistent with hyperactivity problems identified in children exposed to opiates in utero [88].

In an oxycodone self-administration study, offspring exposed in utero demonstrated region-specific effects of oxycodone exposure on MOR-1 at P1; specifically, MOR-1 expression was significantly decreased in the midbrain and forebrain of exposed females [89]. Further, the number of ultrasonic vocalizations by pups exposed to oxycodone in utero varied over time depending on oxycodone intake and pup development. For example, on P3, higher oxycodone intake was associated with fewer vocalizations, while by P9 higher oxycodone intake was associated with a greater number of vocalizations. Maternal oxycodone self-administration and dose-dependent alteration of the maternal-offspring dyad may have resulted in sex-and region-specific effects on early measures of neurodevelopment [89].

Prenatal oxycodone exposure has recently been shown to alter the cargoes of extracellular vesicles (EVs), key players in cell-cell communication [90]. Through RNA-sequencing, distinct miRNA signatures were identified in EVs isolated from P14 rat brains. The gene targets of the dysregulated miRNAs identified in offspring exposed to oxycodone in utero (IUO) or postnatally (PNO) were largely related to the regulation of key functional pathways associated with brain development, with the more impacted group being the IUO. Additionally, treatment of primary neurons with these EVs isolated from IUO brains caused significant reductions in dendritic spine density compared with treatment of PNO or control brain-derived EVs. Dopamine D1 expression was also increased in synaptosomes isolated from P14 IUO brains, potentially a result of the upregulated miR-504 cargo of IUO EVs, which may also play a role in the loss of dendritic spines [90].

The most recent study regarding intergenerational effects of oxycodone exposure continued to explore IUO and PNO exposure groups [24]. Using the F1 and F2 pups descended from oxycodone F0 dams, Odegaard et al. identified physical developmental differences in body weight, head size circumference, and body length. Specifically, F1 IUO pups exhibited lighter body weights at P1, P14, and P30; F1 PNO pups also exhibited this weight deficit at P14 and P30. Interestingly, the F2 IUO and PNO offspring exhibited greater body weights. Additionally, while the F1 IUO and PNO pups had shorter body lengths only at P30, the F2 PNO pups were longer than IUO and controls at P1 and P14. Interestingly, both generations of IUO pups exhibited significantly smaller head size circumferences at P1 [24]. Further, measurements of body mass index and Lee’s obesity index indicated higher percentages of body fat in the F2 generation, particularly in the IUO group. RNA-sequencing conducted on total mRNA isolated from the NAc of P14 pups from each group of each generation identified several differentially regulated genes. Post-validation of these genes revealed alterations in several genes in the orexin/hypocretin system, a system involved in drug addiction and reward-related behavior [91]. Specifically, IUO pups of both generations had higher gene expression levels of hypocretin (*Hcrt*), hypocretin receptor 1 (*Hcrtr1*), neuronal pentraxin 2 (*Nptx2*), and prodynorphin (*Pdyn*) [24]. Furthermore, behavioral studies indicated social deficits in only the F2 IUO pups, and marble burying tests revealed compulsive-like behaviors in IUO and PNO offspring of both generations, with a larger lasting effect seen in the F2 IUO. Taken together, this study found that both in utero and postnatal exposure to oxycodone impacts physical development, behavior, and the expression of key genes involved in addiction in both F1 and F2 generations [24].

## 3. Human Studies

Most human studies regarding generational effects of opioid exposure combine data from different opioid exposure cases together and consider them as one opioid-exposure group. Additionally, most of these studies discuss prenatal opioid exposure and its consequences on the children as they age. Such prenatal exposure studies focus on the immediate outcomes in the F1 children, making a majority of these studies intergenerational. Indeed, short-term consequences of opioid exposure are well-characterized [92], but the long-term, intergenerational consequences require further study. This section will discuss combined opioid exposure studies, where different exposures to morphine, heroin, oxycodone, etc. have been grouped into one opioid exposure group, as well as opioid-specific studies, which consist mainly of heroin with or without polysubstance abuse.

Recently, in utero opioid exposure has been significantly associated with higher risks of fetal growth restriction, preterm birth, lack of normal development, childhood conduct disorder or emotional disturbance in preschool children, and attention-deficit/hyperactivity disorder (ADHD) in older children [93]. A longitudinal study of 8509 mother-child pairs included 454 mother-child pairs exposed to opioids and 8055 control mother-child pairs not exposed to opioids. As most of the sample included urban, low-income, and minority ethnic groups, the higher incidence rates of NAS serve to highlight the plight of prenatal opioid exposure in urban, low-income populations in the inner cities of the United States [93]. Infants born to these mothers had lower gestational age, lower birthweight, and smaller size for gestational age. Within the first six years of age, exposed children had a higher risk of diagnosis with conduct disorders, emotional disturbance, and deficits in physiological development; after six years of age, the likelihood of being diagnosed with ADHD increased in these children. This study adeptly describes the opioid epidemic as an intergenerational problem [93].

A recent meta-analysis of publications spanning from 1993 to 2018 showed the extent of neurodevelopmental outcomes reported in children born to opioid-dependent mothers [94]. Exposed children had lower infant cognitive and psychomotor scores, lower general cognition/IQ and language scores, and higher parent-rated internalizing, externalizing, and attention problems. Overall, this analysis showed that prenatal opioid exposure increases the risk of adverse neurodevelopment in children at least through middle childhood. Important to note, however, is that this meta-analysis only covered three school-aged reported outcomes, limiting the ability to assess longer-term impacts of prenatal opioid exposure [94]. More importantly, this limitation highlights an overall limitation in the field; more longitudinal human studies of prenatal opioid abuse are critical in understanding long-term intergenerational effects of opioid exposure.

In an fMRI study of prenatal drug exposure, which included prenatal exposure to heroin, Geng et al. found that prenatal drug exposure was related to decreased memory performance and altered brain activation during memory encoding [95]. Interestingly, the deficits in memory in adolescents with prenatal drug exposure may stem from variations in encoding rather than in retrieval processes, meaning that potential interventions should focus on encoding rather than retrieval to improve memory performance in these children. This study conducted in 5- to 14-year-old children showed that prenatal exposure to drugs, heroin among them, has long-term effects on memory [95]. Interestingly, a 2010 neuroimaging study found that prenatally-exposed children had smaller intracranial and brain volumes as well as thinner cortex of the right anterior cingulate and lateral orbitofrontal cortex. The volumes of the anterior cingulate, orbitofrontal cortex, and accumbens area were associated with cognitive ability and behavior problems [96].

Providing a paternal/maternal consideration of intergenerational effects beyond prenatal exposure, a recent study by Griesler et al. investigated the intergenerational patterns of nonmedical prescription opioid (NMPO) use in adolescents (12- to 17-year-olds) and their parents. Maternal use of NMPO had a stronger association with adolescent use than paternal use [97]. Use of other drugs also affected both parental and offspring NMPO use. In addition to parental drug use, the quality of the parent-child relationship, adolescent drug use of non-NMPO substances, risk of drug use, drug use by peers, depression, and delinquency were each associated with adolescent NMPO use. These authors therefore suggested that parent-based interventions targeted at NMPO use among youth should not only address parental NMPO use but should also promote positive parenting practices, such as monitoring and reduced conflict [97]. In a similar study considering prescription opioids, Kerr et al. found that paternal opioid misuse and maternal opioid use were associated with the child’s substance use; further, parental prescription opioid misuse or use also predicted child use of alcohol, marijuana, or tobacco by adolescence [98].

In an older study that assessed attentional performance and cardiac reactivity to attentional demand, boys (aged 7 to 12) who were exposed to opioids in utero were compared to boys whose mothers began using illicit substances after the child’s birth (environmental controls), and boys whose mothers were non-drug users [99]. Vagal tone, a measure of heart-rate variability, was measured during an attention task. When distractors were added to the task, opioid-exposed boys failed to suppress vagal tone. Interestingly, both groups with parental opioid exposure made fewer correct responses on this task, indicating that environmental influences, in addition to prenatal opioid exposure, impact attentional performance. The authors concluded that normal physiological responses to increased attentional demand may be impaired in opioid-exposed male children [99].

Of the opioids discussed in this review, heroin appears to be the most studied in human subjects. Illicit heroin use during pregnancy has been associated with several adverse effects in mothers and infants [94]. Prenatal exposure to heroin resulted in reduced head circumference, birth length, and birth weight [100], but it has not been associated with congenital malformations [101]. Additionally, one small retrospective study showed that 93.9% of heroin-exposed infants developed drug withdrawal symptoms within 24 h of birth [102]. This withdrawal, or NAS, may impact language and cognition development [103].

In another interesting study, children (5–6 years of age) born to heroin-dependent mothers were compared to those born to heroin-dependent fathers and three control groups. Out of 83 children born to heroin-dependent mothers, five had significant neurological damage. Of the 76 children born to heroin-dependent fathers, six had significant neurological damage [104]. Further, the children with maternal heroin exposure had lower birthweight and lower head circumference than controls. Parental heroin dependence also resulted in higher hyperactivity, inattention, and behavioral problems in the children. Interestingly, children of heroin-dependent mothers that had been adopted at an earlier age functioned similarly to controls while those raised by their birth mothers functioned significantly lower. This study implicates the role of home environment in the developmental outcomes of children born to heroin-dependent parents [104].

Indeed, prenatal drug abuse, unstable parental care, and low birthweight may contribute to the vulnerability of exposed offspring [26]. A study of 17- to 21-year-old individuals prenatally exposed to heroin and poly-substance abuse revealed lower cognitive functions that appeared to be partially mediated by lower birthweights [26]. Further, the individuals exposed to the least amount of drugs and who had more stable parental care had the best cognitive scores compared to those with unstable homes or mothers who used more drugs during pregnancy [26]. Interestingly, parental factors have previously been shown to play a role in the increased risk of substance use disorders reported in children of heroin-dependent parents [27]. Nygaard et al. continued their investigation of prenatal heroin and polysubstance abuse by studying the mental health of these individuals [105]. Similar to the findings by Vidal et al. [27], a higher proportion of these individuals reported lifetime experiences with major depressive disorder, alcohol abuse, and ADHD [105]. These individuals also scored higher on the aggressive behavior scale and had more sexual partners and were younger at their sexual debut [105].

## 4. Conclusions

Understanding the extent of the opioid epidemic’s effects requires an understanding of multi-, inter-, and trans-generational inheritance. Maternal, paternal, parental, and perinatal opioid exposure also impact future generations in different ways, as evidenced by molecular, developmental, and behavioral alterations in exposed offspring. Morphine, heroin, and oxycodone have all impacted future generations regardless of initial exposure paradigms. These impacts are reported in human and animal models alike.

Animal models of opioid exposure have revealed a number of generational effects in exposed progeny. Molecular alterations in hypothalamic and neuroendocrine systems have been shown to influence sexual maturity, energy homeostasis, and a number of other functions. Dopamine receptor expression levels are also affected by opioid exposure. Further, specific molecular changes in the hippocampus, including variations in LTP and LTD, have been studied, leading to deficits in learning and memory abilities. Behavioral effects also include increase anxiety, opioid tolerance, and aggression. Intergenerational and transgenerational studies provide direction for future epigenetic studies that may further reveal the extent of parental opioid exposure.

Like the animal models of opioid exposure, human studies have reported valuable information about the generational impact of opioid exposure. As many of these studies provide a short-term view of opioid exposure, particularly prenatal exposure, more studies are needed to account for the longitudinal aspect of development. Prenatal opioid exposure has resulted in a number of NOWS/NAS cases, which may impact behavior, as well as physical alterations such as shorter body lengths, lighter bodyweights, and smaller head circumferences. Like in animal models, memory and behavior deficits have been reported in these aging children. Interestingly, parental opioid use has been associated with adolescent use of not only opioids, but of alcohol, tobacco, and marijuana as well. The extent of opioid exposure’s impact on future generations may result from environmental factors; therefore, treatment and prevention for these individuals may need to focus on the parents and home life. Overall, more studies are needed to promote these works from intergenerational studies to transgenerational studies to better understand long-term effects of opioid exposure in humans.

In conclusion, opioid exposure has generational impacts that result in molecular, developmental, and behavioral alterations in future generations. Animal and human models have shown similar results regarding prenatal opioid exposure and its effect on behavior and development. At this time, animal models provide insight into the transgenerational effects of opioid exposure while human studies continue to provide short-term information on intergenerational effects. As the opioid crisis continues and human studies can extend longitudinally, we will likely continue to amass information and extend these works to study transgenerational impacts, allowing us to develop better interventions and treatments over time.
